# Near-Infrared-Triggered Upconverting Nanoparticles for Biomedicine Applications

**DOI:** 10.3390/biomedicines9070756

**Published:** 2021-06-29

**Authors:** Manoj Kumar Mahata, Ranjit De, Kang Taek Lee

**Affiliations:** 1 Department of Chemistry, Gwangju Institute of Science and Technology (GIST), Gwangju 61005, Korea; deranjit@postech.ac.kr; 2 Department of Life Sciences, Pohang University of Science and Technology (POSTECH), Pohang 37673, Korea

**Keywords:** upconversion luminescence, biomedicine, optogenetics, bioimaging, nanoparticles, oncotherapy, biosensors

## Abstract

Due to the unique properties of lanthanide-doped upconverting nanoparticles (UCNP) under near-infrared (NIR) light, the last decade has shown a sharp progress in their biomedicine applications. Advances in the techniques for polymer, dye, and bio-molecule conjugation on the surface of the nanoparticles has further expanded their dynamic opportunities for optogenetics, oncotherapy and bioimaging. In this account, considering the primary benefits such as the absence of photobleaching, photoblinking, and autofluorescence of UCNPs not only facilitate the construction of accurate, sensitive and multifunctional nanoprobes, but also improve therapeutic and diagnostic results. We introduce, with the basic knowledge of upconversion, unique properties of UCNPs and the mechanisms involved in photon upconversion and discuss how UCNPs can be implemented in biological practices. In this focused review, we categorize the applications of UCNP-based various strategies into the following domains: neuromodulation, immunotherapy, drug delivery, photodynamic and photothermal therapy, bioimaging and biosensing. Herein, we also discuss the current emerging bioapplications with cutting edge nano-/biointerfacing of UCNPs. Finally, this review provides concluding remarks on future opportunities and challenges on clinical translation of UCNPs-based nanotechnology research.

## 1. Introduction

Light-emitting nanoparticles have been contributing significantly to the areas of biology and biomedical research for a long time [1,2]. For example, luminescent probes are employed for localization of protein and monitoring biological processes [3,4]. The quantum dots and downconversion nanomaterials which are being used in-vivo biological applications [5,6] fail in terms of penetration depth in the bio-specimens and the common drawbacks of these materials are the scattering of light, low intensity, high photo-damage, poor photo-stability and background autofluorescence which make them unfeasible to meet the challenges of frontier biological science. Therefore, developing novel nanophotonic systems with enhanced features such as deep tissue penetration, less cytotoxicity and low cell-damage for diverse biomedical applications are urgently necessary.

Rare earth (RE)-doped UCNPs can convert low-energy photons into high-energy photons though successive absorption and can overcome many drawbacks that is encountered due to short-wave (ultra-violet or visible) excitation [7,8]. Photon upconversion takes place in RE-doped inorganic materials, where the RE ions act as luminescent centers. It is worth mentioning that upconversion is obtained using low-intensity, low-cost, and readily accessible lasers and has benefits over second harmonic generation and two-photon absorption where high-power and expensive laser sources are required.

In the early 2000s, with the advances in nanotechnology for the preparation of small, high-quality and bright nanoparticles, the studies on the biological applications of UCNPs were initiated. The excellent light emission characteristics such as tunable excitation dynamics, a large anti-Stokes shift, sharp emission bands, etc., are preferable for pioneering nanomedicine platforms. Following this, UCNPs are widely employed in various advanced biological applications, extending from background noise-free biosensing, precision nanomedicine, deep-tissue imaging, to cell biology, visual neurophysiology, and optogenetics [9,10,11,12,13,14,15]. Recently, with our comprehensive knowledge of the upconversion kinetics, it has become possible to engineer the shape and size of the nanoparticles for precise photoluminescence properties. For instance, by synthesizing core multi-shell nanostructures and by selecting the concentration and type of the RE dopants, the wavelength of the emission peaks and their relative intensity can be manipulated [16]. Moreover, upon two separate NIR excitations, orthogonal emissions can be achieved from the UCNPs.

It is interesting that, due to the ladder-like energy levels of the REs, certain ions can even exhibit efficient upconversion in the second NIR window. Thus, it is possible to apply the UCNPs for newer biological applications, including intracellular signaling and deep-tissue bioimaging using NIR-II [17,18]. Although some literature reviews on advances of the synthesis and general applications of UCNPs are already present, in view of biomedicine applications, a focused review on the recent breakthroughs in this field is necessary. Therefore, this article covers the current progress in biomedicine application of UCNPs along with a discussion on the real challenges and perspectives.

In this review paper, we start with the basic knowledge of photon upconverting nanoparticles, including the mechanisms for upconversion luminescence. The progress of UCNPs in biomedicine applications, such as optogenetic modulation, immunotherapy, drug-delivery, photodynamic/thermal therapy, bioimaging and biosensing, is then reviewed thoroughly. The conclusion, future perspectives and challenges for clinical translation have also been discussed in the last section of this review article.

## 2. Basic Knowledge on Upconverting Nanoparticles

Photon upconversion was initially introduced by physicist Nicolaas Bloembergen during the development of infrared photon detectors in 1959 [19]. In the next year, F. Auzel noticed IR to visible photon conversion and unraveled the upconverted light emission processes for Yb^3^^+^–Er^3^^+^ and Yb^3^^+^–Tm^3^^+^ systems. Later in 2004, he summarized the upconversion mechanism in detail [20]. In the very beginning of photon upconversion, the promising applications of UCNPs were not realized. With vast progress in technology, the upconverting materials have been re-investigated in the present century. Photon upconverting materials are usually composed of inorganic host materials doped with a low amount of RE ions. Choosing an appropriate host material is vital for constructing high-quality UCNPs with good optical properties, including high quantum efficiency and a controllable emission profile. Halides (Br, Cl, and I) are considered as ideal luminescent hosts and exhibit low phonon energies (˂300 cm^−1^). However, their hygroscopic nature limits the application. In general, the host materials require a close lattice-matching condition with the dopant ions. The selection of RE is important to regulate the energy transfer within the activator and sensitizer REs. The RE elements have an identical electronic configuration [Xe]4f^n^^−1^5d^0^^−1^6s^2^ that facilitates multiple intra-configurational transitions. The electrons in the 4f-shell RE^3^^+^’s is shielded by totally filled 5s^2^5p^6^ subshells, making them insensitive in the host matrix.

However, all the host materials do not exhibit intense upconverted light emission mainly due to their different co-ordination, RE distribution and energy transfer [21,22]. Low phonon energy materials are usually favored as a host matrix because of the involvement of lesser non-radiative rates, which do not decrease the light emission efficiency. Therefore, particular attention should be paid to the choosing of host, REs and their doping concentration for synthesizing efficient UCNPs.

The phonon energy of the fluoride host materials with formulae NaXF_4_, where X = Y, Gd, La, are efficient host matrices for RE dopants. Moreover, a good chemical and thermal stability of the fluoride nanomaterials is beneficial [23,24]. Within the NaXF_4_ series, hexagonal crystals are more effective than the cubic crystals. This is due to the fact that the probability of Laporte-forbidden intra-4f transitions is higher in a hexagonal crystal due to the less symmetrical crystal field surrounding the REs eventually increasing. Recently, the upconversion from vanadates (GdVO_4_, LaVO_4_, YVO_4_), phosphates (YPO_4_, GdPO_4_, LuPO_4_), and oxides (ZrO_2_, Y_2_O_3_), etc., suggest that they are also efficient materials even though they have a slightly higher phonon energy [23].

The local environment of the RE ions inside a host lattice can monitor luminescence properties. Therefore, to construct effective UCNPs, the major precondition is to have low non-radiative decay rates, long-lived metastable states, a high level of population of the excited states and an even distribution of dopant ions. The luminescence-quenching processes reduce the effective excited states and degrade the photon emission property. It has been suggested that non-radiative transitions are related with the existence of impurities and the presence of RE ions in the surroundings. On the other hand, phonons can bridge the energy states only when the energy gap between the states can be mediated by ~5–6 phonons [25,26]. If the energy gap is higher and it requires more phonons to bridge, then the radiative transitions are dominant. However, in view of the reported results, it is said that the host matrices with Y^3^^+^, Na^+^ and Ca^2^^+^ as one of their elements are commonly used in upconversion systems [27,28,29]. There are also some other factors such as phonon-mediated energy transfer, surface defect density, surface effects, surfactants and surrounding solvents that are considered responsible for luminescence quenching [30].

Another interesting approach to enhance the upconversion intensity is to attach noble metal (e.g., Au, Ag, etc.) nanoparticles on the surface of UCNPs [31]. This enhancement of intensity is due to the surface plasmon resonance (SPR) which can generate large local electric fields in the metal surface’s vicinity. The coupling of UCNPs with SPR can amplify the electromagnetic field acting on UCNPs, and results in an improved upconversion efficiency. The RE^3+^ ions’ electronic transitions and the energy transfer may be affected by SPR under the resonant condition of upconversion emissions with SPR. In core–shell structures, the dopant ions are confined in the interior core of the nanocrystals. The shell can effectively suppress energy loss on the crystal’s surface, leading to an increased luminescence efficiency.

In most cases, Er^3^^+^, Ho^3^^+^ and Tm^3^^+^ ion-doped UCNPs are studied. A list of host materials with RE ions [32,33,34,35,36,37,38,39,40,41,42,43,44,45,46,47,48,49] with their emission peaks upon laser-light excitation is tabulated in Table 1. The energy states of Er^3^^+^—^4^I_15__/2_, ^4^I_13__/2_, ^4^I_11__/2_, ^4^F_9__/2_ and ^2^H_11__/2_/^4^S_3__/2_—can be excited by 800 nm or 980 nm. Likewise, the energy states that take part in Tm^3^^+^ and Ho^3^^+^ are ^3^H_6_, ^3^F_4_, ^3^H_4_, ^1^G_4_, ^1^D_2_ and ^5^I_8_, ^5^I_7_, ^5^I_6_, ^5^F_5_, ^5^F_4_, ^5^S_2_, respectively. It is worth mentioning that there is an upper limit of RE concentration at which the luminescence is highest; thereafter, the emission drops due to a boosted energy transfer among the activators. To overcome the maximum limit of activator ions, the sensitizers are brought into the system so that upconversion is enhanced. The sensitizers offer energy transfer to the activators. The low NIR absorption cross-section of activators reduces upconversion efficiency which can be enhanced by several folds with the help of sensitizers with their wide absorption cross-section at the particular laser excitation wavelength. Yb^3^^+^ is a well-known sensitizer for trivalent REs, including Er^3^^+^, Tm^3^^+^, Eu^3^^+^ and Ho^3^^+^ ions for a larger absorption at 980 nm. The absorption cross-section of the Yb^3+^ ion at 980 nm is 9.11 × 10^−21^ cm^−2^, which is the largest among the RE ions. Moreover, the energy level diagram of Yb^3+^ has only one excited state (^2^F_5/2_) that matches very well with the f–f transitions of many RE activators and, therefore, is an excellent sensitizer to transfer energy to other RE ions. For example, the ^2^F_5/2_ state of Yb^3+^ overlaps the ^4^I_11/2_ state of Er^3+^, allowing a Yb to Er energy transfer.

Other non-rare-earth ions are also doped in UCNPs to tune the upconversion properties; for example, Zn^2^^+^, Na^+^ and K^+^ have shown an improved emission in Y_2_O_3_, BaTiO_3_, CaMoO_4_, etc. [35,40,41,42,43,44]. Following the optimization of the type and number of dopants, UCNPs are coated with polymers to enhance their performance in the surface-bound state [50]. On account of these criteria, it is noteworthy that the selection of dopant ions, their amounts and surface modification must be carefully chosen.

**Figure 1:**
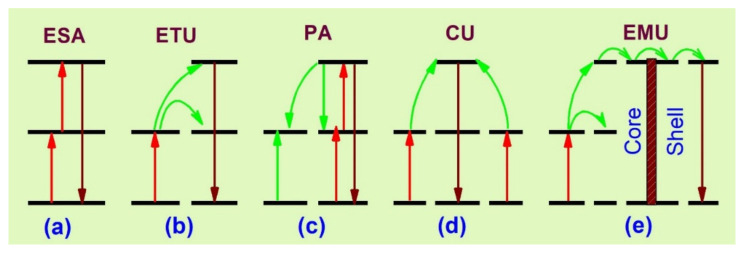
Schematic representation of basic upconversion processes [23]. (**a**) excited stte absorption; (**b**) energy transfer upconversion; (**c**) photon avalanche; (**d**) co-operative upconversion; (**e**) energy migration upconversion.

The five basic upconversion processes [23,51,52,53] are: excited state absorption (ESA), energy transfer upconversion (ETU), photon avalanche (PA), cooperative upconversion (CU) and energy migration-mediated upconversion (EMU). The primary upconversion processes are described in Figure 1.

ESA occurs through the absorption of one or multiple sequential photons from the lower energy state to the intermediate, from which upconversion is obtained (Figure 1a). The excited ions can further absorb another pump photon and it occurs when the activator’s concentration is dense, but the process may decrease the upconversion efficiency by radiationless relaxations. The ETU process (Figure 1b) includes the energy transfer between sensitizer and activator, and it is more efficient than ESA. Once a sensitizer is excited, the transfer of energy to the adjacent activator takes place and luminescence is obtained with the relaxation of the activator ion to a lower state. For an effective energy transfer process, the sensitizer–activator should have resonant and spatially separated energy levels. Thus, because of the resonant energy transfer, the excitation time is slow [54,55,56,57].

The PA (Figure 1c) takes place after a certain amount of pump power density. At a high excitation pump density, the intermediate states of many activators are populated by absorption through the ground state and the upconversion emitting level is populated by ESA or ETU from the other excited ions. Subsequently, a cross-relaxation process occurs between the excited state ion and the ground state ion. This event populates the reservoir and the light-emitting states, resulting in an ‘avalanche’ generation of many excited ions by feedback looping [53]. The CU (Figure 1d) mechanism is mainly involved in Yb^3^^+^/RE^3^^+^. In CU, co-operative energy transfer takes place when the upconversion-emitting level of the activator is populated through two neighboring sensitizer ions. However, the most recently known upconversion mechanism is EMU (Figure 1e), which occurs with the help of four types of ions—sensitizer, accumulator, migrator and activator. In this process, the migrator links the activator and sensitizer/accumulator ions. The role of the sensitizer is to promote the accumulator ion into a higher excited state through energy transfer; the excited accumulator further migrates the energy via the core–shell interface to the migrator after the first event. An activator layer then traps the energy in the shell and an upconversion process takes place.

The surface modification of the RE-doped UCNPs is necessary to develop bioconjugation of the nanoparticles and modification is executed by chemical or physical interaction. As a result, the UCNPs gain functional groups or become strongly charged on the nanoparticle surface and become suitable for conjugation with biomolecules. Surface modification enhances photostability of the nanoparticles and facilitates the platform for conjugating biomolecules for various bio-medicinal applications [58,59,60,61]. There are several conjugation strategies used to modify the UCNPs surface, for example: the ligand oxidation reaction, ligand exchange, layer-by-layer self-assembly process, coating by polymer molecules, etc. The original ligand is displaced by polymeric molecules on the nanocrystal substrate in the ligand exchange method. For example, Chow and his co-workers functionalized the NaYF_4_:Yb/Er nanoparticles by carboxyl groups [62]. The ligand oxidation technique includes a carbon–carbon double bond oxidation by Lemieux-von Rudloff reagent. Usually, the carbon–carbon double bonds are transformed into carboxylic groups to facilitate reactive functional moieties. Chen et al. scaled up a simple strategy for converting hydrophobic UCNPs into water-soluble ones [63]. The oxidized UCNPs can be directly conjugated with proteins in the presence of free carboxylic groups and a DNA sensor was fabricated by this technique. Li et al. reported a layer-by-layer strategy using oppositely charged linear polyions [64]. Usually, oleic acid controls the crystal growth and develops a hydrophobic coating on the surface of the ligands. Subsequently, surface modification is desired to form a hydrophilic surface composition prior to their use in bioanalytical applications. Sometimes, a layer of silica shell makes it a more accessible platform for conjugating various functional groups. On the other hand, binding silver or gold nanoparticles on the surface of UCNPs can facilitate thiol-containing ligands. Several surface modification techniques also depend on the intermolecular interactions of the UCNP surface and the ligand. For instance, the hydrophilic ligands can replace the hydrophobic surface ligands through a ligand exchange reaction. The hydrophobic surface can be oxidized partially to obtain a hydrophilic surface and a further modification is performed to bind biomolecules on the UCNP’s surface. A recent article [61] has excellently reviewed the surface modification of UCNPs in this regard.

## 3. Biomedicine Applications

Biomedicine is a multidisciplinary research field of biotechnology and medicine that is comprised of biomaterials for biological research in medical diagnostics and therapy. Nanoplatforms using UCNPs are useful for non-invasive diagnosis and delivery of therapeutic components that can even evaluate in vivo pharmacokinetics in real time [65,66,67]. To assist medical surgery, the UCNPs can also be used in diagnosis and therapy as a real-time agent to target with the loading of gene, drug or biomolecules at a specific location. The other benefits of UCNP-based theranostics are their minimum side effects as compared to the passive targeting system and enhanced therapeutic effects. The NIR imaging of tumors was presented by Weissleder et al. [68], where the tissue penetration depth was 1–2 cm and this value is deeper than that of visible light. Since then, researchers are developing techniques along with the excitation and emission wavelengths in the NIR window (700–1700 nm). A report shows that due to the low absorption of water at 808 nm, the penetration depth can be as high as 10 times for a depth of 10 mm, where melanin in the skin is likely to reduce the penetration depth [69]. The 650–1350 nm wavelength has a maximum depth of penetration in tissue. Since biological tissues exhibit comparatively low NIR light scattering and attenuation, the penetrating ability of NIR light is deeper than visible light. A list of NIR-responsive UCNPs with their biomedical applications are tabulated in Table 2 [70,71,72,73,74,75,76,77,78,79,80,81,82]. The advances in major biomedicine applications of UCNPs are described below:

### 3.1. Neuromodulation and Optogenetics

**Figure 2:**
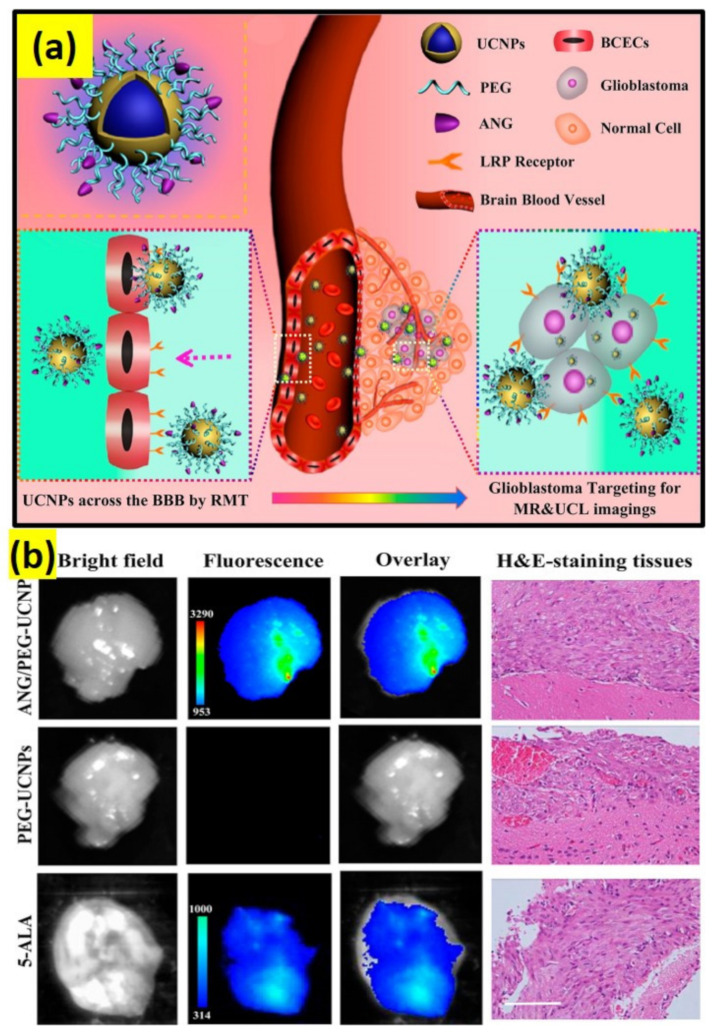
(**a**) ANG/PEG-UCNPs: a dual mode targeting system for BBB crossing and targeting the glioblastoma. (**b**) Glioblastoma-bearing brain images after 1 h of intravenous injection with 5-ALA (excitation, 470 nm; emission, 650 nm) and ANG/PEG-UCNPs, PEG-UCNPs (excitation, 980 nm; emission, 800 nm); scale bar: 100 µm [85].

Brain research is the most complex study, particularly the brain stimulation for manipulation and therapy [83]. Therapy for brain cancer has encountered many hindrances due to the blockage of drugs to the brain–blood–brain barrier (BBB) in general chemotherapy [84]. It is proven that therapeutic agent-loaded nanoparticles have the capacity to cross through the BBB. For example, angiopep-2-functionalized UCNPs can be employed for brain glioblastoma photothermal–photodynamic therapy by facilitating transcytosis (Figure 2a,b) [85]. The currently available radiotherapy can be substituted by optogenetics, where it is possible to achieve neural inhibition. In this way, through an optical signal, the UCNP sensitized with NIR dye can not only extend the absorption range, but also increase the emission efficiency.

For optogenetic inhibition, the core–shell–shell UCNPs were developed to match with the excitation band of the inhibitory opsin protein [86]. In a separate study, dye-sensitized poly(methyl methacrylate) polymer-embedded UCNPs were reported as an implantable scheme with the loading of Pluronic F127 to tune the excitation window to 800 nm for optogenetic neuron excitation so that deep-tissue imaging becomes possible in the mouse model [87]. The manipulation of optical neuronal activity by a minimal invasive approach has reported to speed up optogenetic therapy through wireless optogenetic implementation. In this regard, this crucial work explains that molecularly-arranged UCNPs can act as actuators for deep—brain neuron—stimulation by transcranial NIR light [12].

Transcranial NIR UCNP-assisted optogenetics promote the release of dopamine from the genetically labeled neurons in the ventral tegmental area, trigger inhibitory neurons in the medial septum for inducing brain oscillations, and inhibit excitatory cells of hippocampus for silence seizure and triggering memory recall [12]. Another work shows that through a minimal invasive technique, optogenetic stimulation beyond a 2 mm depth of neurons into brain tissue is possible through UCNPs encased in a Parylene C microstructure, whose blue light excites opsins with a high resolution by irradiating NIR light [88]. Another study illustrates that the implantation of cylindrical pillars of Parylene C polymer can be employed deep into the tissue. The enclosed UCNPs absorb NIR at 980 nm, which is absorbed less than blue light by brain tissue [88]. A separate study reports UCNPs with a photoreceptor for anchoring retinal photoreceptors and sending signals to the brain upon NIR light. Interestingly, this work further shows NIR light visual enhancement in night, enabling mammals to see light over a 700 nm wavelength [89]. 

### 3.2. Immunotherapy

**Figure 3:**
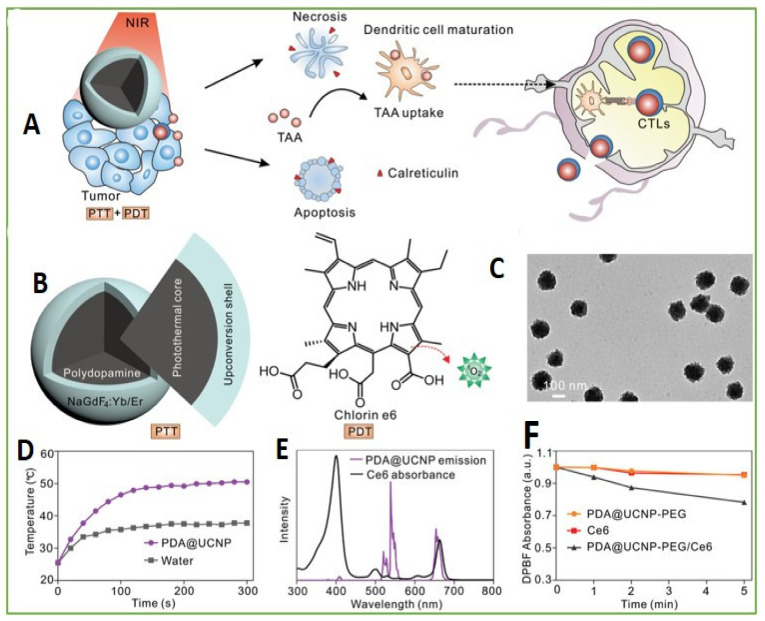
Design of PDA@UCNP-PEG/Ce6. (**A**) Scheme for synergistic phototherapy. Upon laser irradiation, the UCNP-based system can ablate the tumor, tumor-associated antigens (TAA) are released and the antitumor immunity is triggered. Finally, it helps the inhibition of tumor metastasis. (**B**) The UCNP structure: core for PTT and the shell for PDT. (**C**) TEM images of PDA@UCNP. (**D**) Temperature variation with irradiation time of PDA@UCNP nanoparticles (2 mg mL^−1^). (**E**) Ce6 absorption and PDA@UCNP emission upon 980 nm laser excitation. (**F**) ^1^O_2_ generation comparison upon 1 Wcm^−2^ laser irradiation. Adapted with permission from [91].

Immunotherapy is considered potential for antitumor growth and metastasis. This therapy has the ability to stimulate the immune system of the body and, thus, boosts the natural ability of the body to fight cancer. The present immunomodulation system has disadvantages, such as off-tumor toxicity, insufficient immune response, and detrimental autoimmune effects. In this respect, pretreatment of phototherapeutic is usually implemented prior to immunoregulation for enhancing the immune response by delivering antigens associated with tumors and a convenient immunological microenvironment in lesions by the initiatory death of tumor cells. In recent years, a mesoporous silica, combined of an upconversion nanotransducer, vaccine antigen, adjuvant, and photosensitizing molecule, was utilized as an immune activator to enhance cancer immunotherapy and promote immunopotentiation [90]. Yan et al. reported synthesis of upconversion nanoparticles encapsulated by polydopamine for antitumor immunity and antimetastatic activation by photodynamic and photothermal therapeutic methods, through utilizing energy conversion from UCNPs (Figure 3) [91]. 

Another recent study showed NIR-irradiated ROS generation from UCNP systems aimed at activating the immune response for anti-tumor therapy [92]. The study further presents that low-dose ROS can effectively activate T-cell immune responses while the high-dose ROS can cause immunogenic cell death and release tumor-associated antigens. Recently, a demonstration of selective immunomodulation along with spatiotemporal regulation was explored by DNA-conjugated UCNP nanomachines [93], where immunostimulatory agents were encapsulated in a single-strand DNA. Therefore, the UCNP-based immunotherapy gives a remote and noninvasive strategy to regulate adjuvant activity with great spatiotemporal precision for decreasing systemic toxicity. However, these above-mentioned strategies rely on one or several selected tumor-associated antigens, inevitably running the risk of failure to generate an immune response due to tumor heterogeneity.

### 3.3. Drug Delivery

Despite a substantial development in cancer therapeutics, chemotherapy is still favored in clinical anticancer treatment. Although, chemotherapy practice generally includes several demerits such as low drug solubility, elusive dosage, drug tolerance factors, and contraindications. Therefore, a solution to these concerns in chemotherapy with a robust and effective drug delivery technique is essential to precision nanomedicine which should have excellent biocompatibility, be capable of loading therapeutic molecules, have inhibition of premature drug leakage ahead of its stimulus-activation, responsivity to stimuli, and flexibility over the control of drug release. A recent report shows the use of upconversion-induced azo photoisomerization for regulating the release of drug, interposed into DNA helices [94]. In this case, nuclear-targeting peptides with hyaluronic acid were used for the functionalization of the nanoplatform’s surface to obtain a perinuclear aggregation of drugs [94]. In another study, by temperature-feedback photothermal modulation, it was possible to administer a precise drug release through phonon-susceptible upconversion luminescence [95]. The nanoplatform is also suitable for delivering biomolecules—prodrugs, nucleic acids, etc.—and metal ions—Zn^2^^+^, Mn^2^^+^, Fe^2^^+^, etc.—for oncotherapeutics. Additionally, UCNP-based drug delivery can also be employed for gene delivery. A remote-operative gene editing nanoplatform based on NIR modulation of UCNPs was also developed for release of CRISPR-Cas9 in deep tissue [96], though such an upconversion-derived CRISPR strategy is a matter of concern in terms of in vivo gene editing efficacy, delivering system’s physicochemical stability, stimulatory signal’s penetration depths, etc.

**Figure 4:**
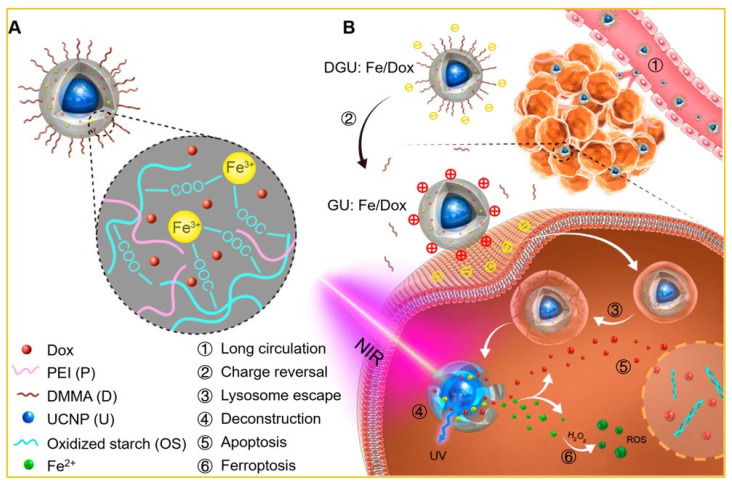
(**A**) Fe^3^^+^ -linked carrier: UCNP (core) and Dox (absorbed in the polymer shell). (**B**) Anticancer mechanism of the UCNP-based system. ① Passive accumulation of DGU:Fe/Dox with extended circulation and enhanced EPR. ② Change from DGU:Fe/Dox (negative) to GU:Fe/Dox (positive) at tumor site driven by pH activation. ③ Lysosome escape of GU:Fe/Dox through proton sponge. ④ Deconstruction of NIR-responsive system under the action of UCNP. 2464 Apoptosis of released Dox in the cell nucleus. ⑥ Ferroptosis of ROS with tumor cellular H_2_O_2_ at the cytoplasm. Adapted with permission from [100].

Drug release has been exclusively illustrated with doxorubicin (Dox), which is a front-line drug in chemotherapy, but the use of Dox is challenging due to unwanted side-effects and non-specific damage to normal cells. A number of reports are available on Dox release on this ground [97,98]. UCNPs with Fe(OH)_3_ were used to generate additional ROS creation along with OH free radical and H_2_O_2_ via a Fenton-like reaction. Through this chemical reaction, Fe^3^^+^ was introduced to Fe^2^^+^ by absorbing UV light emitted from Fe(OH)_3_-UCNP upon IR irradiation [99]. These UCNPs were further functionalized by a pH probe and Dox for sensing-guided therapy on the basis of simultaneous application of chemotherapy and PDT. The nano-transport system was coated with 2,3-dimethyl maleic anhydride (DMMA) and PEI and an extended drug circulation time with a fast release after surface modification was observed (Figure 4) [100].

Another study grafted photoactivatable Pt(IV) prodrugs onto core–shell UCNPs of NaYF_4_:Yb^3^^+^/Tm^3^^+^@NaGdF_4_:Yb^3^^+^ for phototherapy. In this case, the drug release occurred by NIR light irradiation following the delivery of nano-cargo into cancer cells through the endocytosis process. Moreover, owing to the smaller size, the UCNPs successfully internalized in cells avoiding biological barriers and showed extended blood circulation for in vivo works. The UCNPs escaped from the tumor within 1 h and released the drugs for chemotherapy followed by NIR-driven dissociation [101]. 

### 3.4. Photodynamic and Photothermal Therapy

#### 3.4.1. Photodynamic Therapy

Photodynamic therapeutics (Figure 4) work on the principle that the generated ROS causes apoptosis of cancer cells. It has three main elements: excitation light, photosensitizers, a and source of oxygen. A photosensitizer at the ground state is raised into a high-energy state after absorption of light. Thus, an energy transfer takes place to the neighboring oxygen molecules. This process generates singlet oxygen (^1^O_2_) or other ROS. The produced ROS species then destroy the tumors by multifactorial mechanisms, such as the necrosis and/or apoptosis process inducing cancer cell death, an anti-angiogenesis effect destroying the tumor vasculatures, etc. The main limitation of using only a photosensitizer is that it requires high-energy light (visible, UV light) to be activated, but these high-energy lights have a very poor tissue penetration ability in biological tissues, hampering the treatment of internal and large tumors through PDT. To solve this issue, NIR light-sensitive UCNPs are used. It is also considered that NIR light has a low phototoxicity compared to normal cells and, thus, the UCNPs are ideal for PDT [102].

The intelligent design and surface engineering of UCNPs can not only address this issue by using NIR light, but also include features to counter the tumor microenvironment, such as hypoxia. Photoconversion efficacy can also be enhanced by designing multi-layered core–multishell nanostructures, avoiding surface quenching and concentration effects. Multilayered, core−shell nanostructures are usually employed to combat the concentration and surface quenching effects. Sometimes, dyes or additional lanthanides are used as photon absorbers to overcome the overheating issue from laser excitation sources and, thus, it improves therapeutic performance. A recent study revealed the self-assembling of UCNP-conjugated pH-responsive polymer molecules for deep tissue ROS generation and amplification of therapeutic efficacy through pH activation [103]. In another study, covalent cross-linking within 2-cyanobenzothiazole and exposed cysteine on UCNPs was introduced by the cleavage of peptides through protease and, thus, promoted singlet oxygen generation due to an enhanced photosensitization [104]. Various ligands and antibodies conjugated with UCNPs have also been used for targeting tumors and photodynamic nanotheranostics. Recently, it has been demonstrated that an assemble of several building blocks in a single platform can induce a cascade reaction, e.g., UCNPs, Au, MOF (metal organic frameworks), and can generate O_2_, H_2_O_2_ and ^1^O_2_ in therapeutic dynamics [105]. However, UCNP-based PDT still has prominent challenges, such as poor efficacy for big tumors, damage of normal cells due to laser irradiation and limitation for treating metastate tumors.

#### 3.4.2. Photothermal Therapy

**Figure 5:**
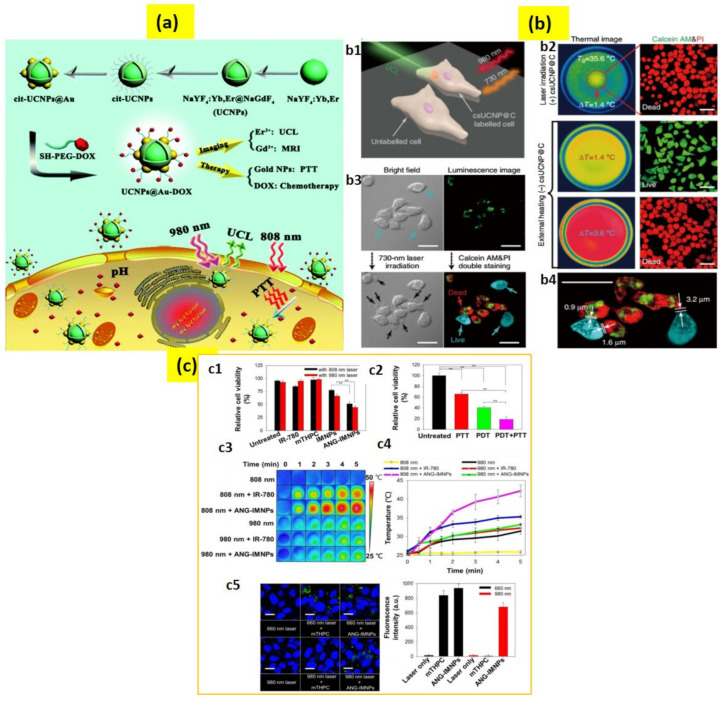
(**a**) Schematic presentation of the UCNPs@Au-DOX synthesis, luminescence (UCL)/magnetic resonance (MR) imaging, photothermal therapy (PTT), and chemotherapy. (**b**) High-accuracy PTT using csUCNP@C. (**b1**) Schematic illustration of PTT. (**b2**) Images of HeLa cells with photothermal ablation (**b3**) PTT of HeLa cells upon 730 nm light excitation for 5 min. csUCNP@C-labeled cells exhibited a strong upconversion signal in the cytoplasm (green). (**b4**) Amplified image of (**b3**). (**c**) PDT/PTT effects on ALTS1C1 cells. (**c1**) Cytotoxicity of mTHPC, free IR-780, IMNPs, and ANG-IMNPs upon 808 nm or 980 nm laser light. (**c2**) Cytotoxicity of PTT and PDT; symbols and error bars are mean ± S.D. ** *p* < 0.01. (**c3**) Thermal images and (**c4**) temperature rise profiles upon irradiation. (**c5**) Laser scanning confocal microscopy images and the corresponding quantitative comparison of in vitro ROS generation in ALTS1C1 cells; singlet oxygen sensor green staining shown in green; blue shows cell nuclei stained with DAPI; scale bar: 20 μm. The 660 nm light generated due to upconversion reaction of the NIR laser inside the cancer cells, increasing fluorescence intensity. Adapted with permission from [85,111,114].

The working principle of photothermal therapy (PTT) is comparable to PDT, but the difference is that in PTT, photon energy is converted into local heat energy via phonon–phonon coupling [106,107]. and the technique is promising for cancer treatments. For instance, Ho^3^^+^/Tm^3^^+^-doped KLu(WO_4_)_2_ UCNPs were used to obtain a controllable release of thermal energy for transferring heat in the nano regime [108]. In another study, a theranostic nanoplatform combining PTT of cancer and upconversion imaging was integrated by designing a nanocomposite of nanographene oxide and NaLuF_4_:Er^3+^,Yb^3+^ UCNPs [109]. A dual-mode system for image-guided PPT comprised of UCNPs coated with a cancer cell membrane and gold nanoparticles was recently developed for high efficacy PTT therapy [110]. Another study presented a novel method for chemotherapy and PTT using the in situ growth of gold nanoparticles onto the citric acid functionalized UCNPs (cit-UCNPs@Au). Here, a chemotherapeutic agent—SH-PEG-DOX—prodrug was bonded with cit-UCNPs@Au to design a nanocomposite of UCNPs@Au-DOX (Figure 5a) [111]. A hydrazone bond in SH-PEG-DOX was introduced for pH-triggered drug release in the cancer cell microenvironment [112].

In another major study, carbon-coated UCNPs of a core–shell structure (NaLuF4:Yb,Er@NaLuF4@Carbon) were developed by Zhu et al. [113], where the outer shell of carbon acted as a superior PTT agent upon 730 nm light excitation and have the capability to kill HeLa cancerous cells without damaging the neighboring normal cells (Figure 5b) [114,115]. To increase PTT, monosaccharide UCNPs have proven to cause significant photothermal damage to the cancer cells compared to the normal UCNPs due to the higher attraction of monosaccharide by the cancer cells [116,117]. An image-guided combinational approach for PDT and PTT is also a considerable strategy for the UCNP-based therapeutic system [118]. For example, an efficient dual-mode UCNP system for PDT/PTT could selectively deliver a photosensitizer into the tumors of a brain astrocytoma and a substantial enhancement of the median survival rate of the tumor was observed (Figure 5c) [85]. Apart from the above reports, micelles composed of UCNPs conjugated with a photosensitizer, antibody anti-EpCAM, and mitoxantrone (MX) anti-cancer drug, were used for targeting cancer stem cell biomarkers to synergetically lower cancer recurrence, prognosis, and metastasis [119]. Alternately, PTT for bacterial killing was performed by employing Nd^3+^/Yb^3+^-doped Y_2_O_3_ [106]. However, due to the huge amount of heat generated it is applicable only for the in vivo environment.

### 3.5. Near-Infrared Sensitive Bioimaging

The conventional bioimaging probes are fluorescent proteins or organic fluorophores, but they generate a strong background signal and have a low photostability. The limitation of photostability can be overcome by quantum dots but they suffer from a toxicity issue. On the other hand, UCNPs have excellent chemical properties, a long penetration depth, no background noise and an intense upconversion emission, making them superior biomarkers over fluorophores and quantum dots [120,121,122,123,124]. Most of the UCNPs are doped with Yb^3^^+^ and Nd^3^^+^ sensitizers and Er^3^^+^, Ho^3^^+^ and Tm^3^^+^ activators, mainly because of their suitable energy levels which can be excited by 976 or 808 nm light and upconversion emission in a broad range spanning from UV to NIR [125,126]. However, using 808 nm laser light for excitation has a major benefit over using 976 nm in terms of suppressing overheating [127,128]. The excitation wavelength can also be broadened for in vivo imaging up to 1530 nm through the sensitization of Er^3^^+^ ions [129]. It is to be mentioned that with the rational design of the UCNPs, a nanoparticle has the capability to be excitable at multiple wavelengths, resulting in upconversion emission at various wavelengths and, thus, orthogonal upconversion luminescence is achieved in a single UCNP as an all-in-one platform for biological applications [130]. The only disadvantage of UCNPs is that the quantum yield of photon upconversion is comparatively low; various approaches are being considered now a days to counter this issue.

**Figure 6:**
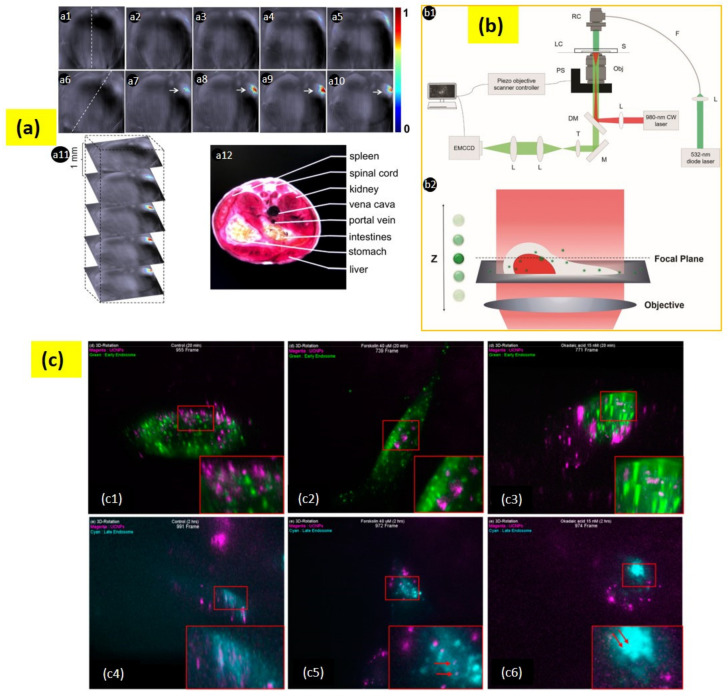
(**a**) (**a1**–**a5**) Live mouse anatomy sections prior to injection of UC-α-CD. (**a6**–**a10**) Anatomy sections after 35 min post-intravenous injection of UC-α-CD. Figure (**a1**,**a6**) with a dashed line indicates the mouse position. (**a7**–**a10**) suggests UC-α-CD localization. (**a11**) Three-dimensional image collection. (**a12**) Analyzed area related section. (**b**) (**b1**) The setup for a wide-field epi-fluorescence microscopy. The 980 nm CW laser is the excitation source for UCNPs; the 532 nm diode laser is the light source for RFP; the acronyms used in the diagram are as follows:RC—reflective collimator; LC—live-cell chamber; S—sample; F—optical fiber; PS—piezo objective scanner; Obj—objective lens; L—lens; DM—dichroic mirror; T—tube lens; M—mirror. (**b2**) Scheme for scanning of the objective lens. (**c**) Early state colocalizations of UCNPs with early endosomes or late endosomes in tau aggregated SH-SY5Y cell. (**c1**) Control 20 min (955 frame), (**c2**) forskolin 20 min (739 frame), (**c3**) okadaic acid 20 min (771 frame), (**c4**) control 2 h (991 frame), (**c5**) forskolin 2 h (972 frame), and (**c6**) okadaic acid 2 h (974 frame). Magenta: UCNPs; green: early endosome; cyan: late endosome. Adapted with permission from [134,141,142].

A primary report by Chatterjee et al. presented PEI modified NaYF_4_:Er^3+^,Yb^3+^ nanoparticles for cancer cell imaging, where a strong green upconversion emission was observed upon NIR laser excitation [131]. In 2012, Liu et al. internalized PEG-coated NaYbF_4_:Er^3^^+^, Gd^3^^+^ UCNPs into HeLa cells and compared images of rat hearts with the developed UCNPs and clinically used a contrast agent—iobitridol. Although their concentration was the same, the UCNPs showed better contrast images than that of iobitridol [132]. The UCNPs can also be used for computed tomography (CT) imaging. For this purpose, UCNPs should be attached with some compounds that can absorb X-ray radiation. For example, iodine compound-conjugated UCNPs were employed for CT imaging of the liver for 30 min and it exhibited an extended circulating time [133]. UCNPs have also been found to be suitable for photoacoustic imaging. Maji et al. used water-soluble α-cyclodextrin-covered NaYbF_4_:Er^3^^+^, Yb^3^^+^ for photoacoustic imaging based on a non-radiative process-led quenching of photoluminescence, resulting in an improvement of the photoacoustic signal under 980 nm excitation [134]. To study its capability for photoacoustic imaging, in vivo localization of the complex was illustrated in live mice (Figure 6a). Additionally, UCNPs have been explored as MRI contrast agents. Most of these studies contain Gd^3^^+^ ions in the UCNP’s host or dopant. For instance, NaGdF_4_ NPs were utilized for atherosclerotic plaque imaging and MRI angiography, and showed that UCNPs are more efficient than some MRI contrast agents available commercially [135]. Furthermore, multiple bioimaging techniques can be combined for developing multimodal bioimaging [136,137,138,139].

Our group is constantly developing a three-dimensional bioimaging technique for high spatiotemporal resolution using UCNPs and their cellular tracking [140,141,142,143]. Recently, we reported a three-dimensional live-cell imaging by collecting two-dimensional section images for understanding cellular dynamics [140]. Later, we further integrated the experimental set-up to quantitatively evaluate the cellular uptake efficiency of various chemical and biological functional group-conjugated UCNPs with their three-dimensional information (Figure 6b) [141]. Recently, we studied PAA-conjugated UCNPs for an intracellular transport mechanism through the internalization of the nanoparticles in tau aggregated neuroblastoma cells (Figure 6c) [142]. 

### 3.6. Upconverting Nanoparticles for Biosensing

#### 3.6.1. Biomolecules Sensors

**Figure 7:**
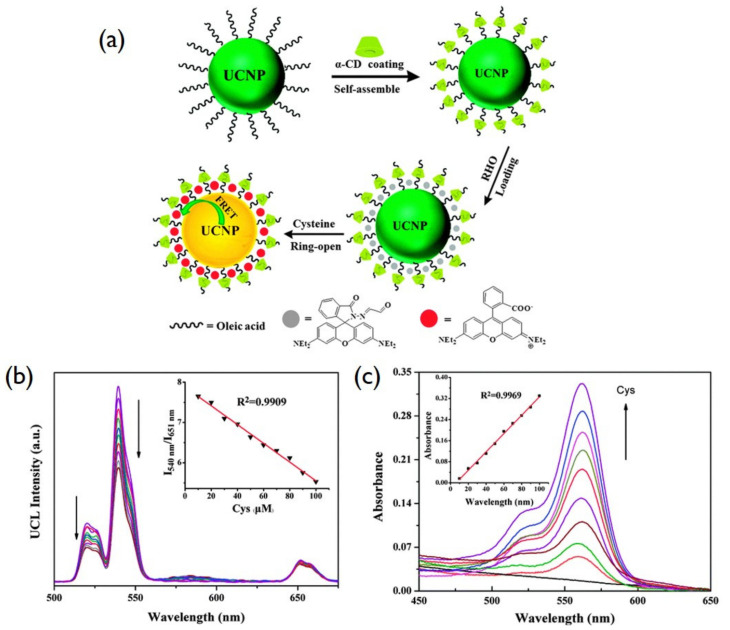
(**a**) Schematic description of the UCNP surface modification and FRET based on donor UCNPs and acceptor rhodamine. (**b**) UC emission spectra under 980 nm, (Inset: the variation of relative UC emission intensity at a 540 nm to 651 nm ratio upon different amount of Cys. (**c**) UV–Vis absorption titration spectra (Inset: the linear response of the absorption peak intensity at 562 nm and Cys concentration) of RHO functionalized UCNPs with a gradual increment of Cys. Reproduced with permission from [144].

Biomolecules sensors are usually based on the FRET (fluorescence resonance energy transfer) technique, in which a donor’s fluorescence can be successfully quenched by the acceptor. Wang et al. designed a FRET with UCNPs as donors and gold nanoparticles as acceptors to detect trace amounts of avidin [143]. In that work, gold nanoparticles (GNPs) were employed as the UCNPs’ quencher. Another study on the combining of a cyclodextrin-coated upconversion luminescence platform for detection of cysteine was carried out by Ni et al. [144]. The work used the novel rhodamine-oxaldehyde (RHO)-functionalized β-NaYF_4_:Yb^3^^+^/Er^3^^+^ to detect cysteine in an aqueous solution. In this case, β-NaYF_4_:Yb^3^^+^/Er^3^^+^ was used as an acceptor and rhodamine-oxaldehyde as a donor (Figure 7).

**Figure 8:**
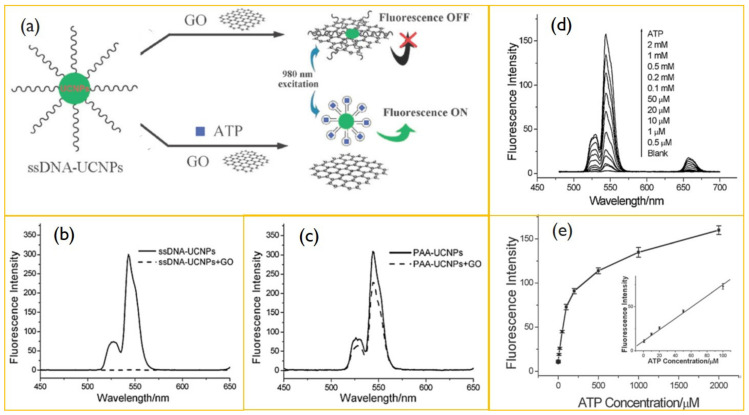
(**a**) Schematic illustration of ATP sensing using a resonance energy transfer between ssDNA-UCNPs. (**b**) Upconversion spectra of ssDNA-UCNPs in Tris-HCl buffer after (dashed line) and before (solid line) incubation with GO. (**c**) Upconversion emission of PAA-UCNPs in Tris-HCl buffer after (dashed line) and before (solid line) the addition of GO. (**d**) Upconversion spectra of UCNPs-GO in the presence of 0–2 mM ATP. (**e**) Plot of upconversion emission intensity (at 547 nm) vs. ATP concentration. Reproduced with permission from [145].

With the increase in the cysteine concentration, the quenching of luminescence was found to increase under NIR excitation. Liu et al. presented that water-soluble graphene oxide (GO) can quench the fluorescence of single-stranded DNA-functionalized UCNPs, where β-NaYF_4_: Yb, Er nanoparticles are used as the UCNPs donor [145]. Under 980 nm excitation, the ssDNA as adenosine triphosphate-specific aptamer exhibits a strong upconversion luminescence which can be quenched by the addition of GO. Meanwhile, with adenosine triphosphate, the luminescence was recovered (Figure 8). The study further proposes that the UCNPs-GO FRET assay can be used to detect various target molecules by cross-linking the specific aptamers with the UCNPs. Moreover, UCNP-based sensing is effective for the detection of tyrosine working on the principle of a photoinduced electron transfer mechanism between NaYF_4_:Yb/Tm nanoparticles and melanin-like polymers [146]. Additionally, Guo et al. developed an upconversion-based detection system for glycoprotein [147]. These works suggest that UCNPs can be applied for the detection of biomolecules in a range of chemical and biological analyses due to an improved signal to noise ratio, a high resistance to photobleaching, narrow emission bands, and deep tissue penetration depths [148]. 

#### 3.6.2. ROS (Reactive Oxygen Species) Sensors

**Figure 9:**
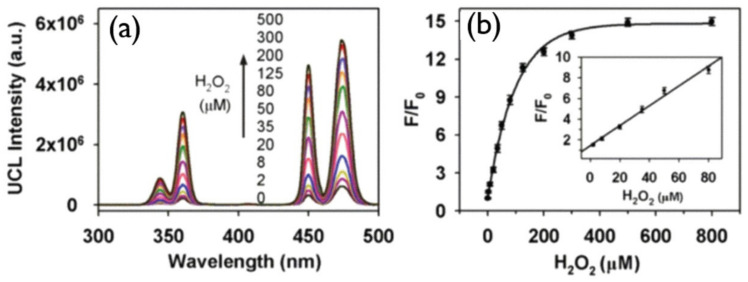
(**a**) Upconversion spectrum of DNA-AgNPs/UCNP at various concentrations of H_2_O_2_. (**b**) 450 nm emission enhancement (F/F_0_) on increasing the amount of H_2_O_2_ in DNA-AgNPs/UCNP; F and F_0_ correspond to the upconversion emission intensity in the presence or absence of H_2_O_2_, respectively, in the system. Adapted with permission from [149].

ROS have significant effects in cell signaling and homeostasis and a certain level of ROS can damage cells, DNA, RNA, lipid peroxidation, and amino acids oxidation. Therefore, knowing the exact concentration of ROS is vital for biosystems. They are formed as natural byproducts of normal metabolism. Some of the examples of reactive oxygen species are H_2_O_2_, HClO, and OH. To detect H_2_O_2_ and glucose, Wu and his group fabricated a LRET-based upconverting hybrid nanocomposite [149]. In this work, the nanocomposite was developed by combining DNA-templated silver nanoparticles (DNA-AgNPs) and NaYF_4_:Yb/Tm@NaYF_4_ UCNPs where DNA-AgNPs acted as a quencher and NaYF_4_:Yb/Tm@NaYF_4_ as a donor. A cytosine-rich DNA sequence was employed for the nucleation of AgNPs and a poly (adenine) sequence was attached at the 3′ end of the C-rich sequence to increase the accessibility to the UCNPs. Based on the coordination interactions between the DNA-AgNPs’ negatively charged phosphonate groups and the exposed REs on UNCPs, DNA-AgNPs could assemble on the surface of UCNPs to form a DNA-AgNPs/UCNP nanocomposite. This design worked on H_2_O_2_ modulation of the DNA-AgNPs-induced luminescence quenching of UCNPs (Figure 9a).

Spectral overlap between the blue emissions of UCNPs and the absorption of DNA-AgNPs was observed in the absorption spectrum of DNA-AgNPs and upconversion emission of NaYF_4_:Yb/Tm@NaYF_4_; as a result, upconversion luminescence was quenched by DNA-AgNPs, as shown in Figure 9b, and with the addition of H_2_O_2_ the luminescence was recovered. In [150], Chu and co-workers put forward a manganese dioxide-modified UCNP sensor for the detection of H_2_O_2_ and glucose. This design is based on H_2_O_2_ modulation of the MnO_2_^-^-induced luminescence quenching of UCNPs. Upconverted luminescence of UCNPs was quenched by the addition of H_2_O_2_, which reduced MnO_2_ to Mn^2^^+^, and the glucose can be identified on the basis of glucose oxidase for generating H_2_O_2_. 

In addition, OH radicals can damage biomolecules in living systems; therefore, monitoring OH radicals is necessary to understand their physiological roles. In this regard, Li and co-workers developed a UCNP probe for the detection of OH radicals [151]. It shows that upconversion emission from the UCNP is suppressed due to carminic acid and can be recovered by the addition of OH. A highly sensitive multifunctional probe has been reported recently to detect OH concentration [152]. Hypochlorous acid (HOCl) also plays a role as a key microbicidal agent for immune systems but its misproportion could damage the tissues. Zhang and co-workers reported a UCNP-based probe for ratiometric detection of HClO [153], where a water-soluble nano detection system containing rhodamine-modified UCNPs was synthesized.

#### 3.6.3. Intra-Cellular pH Sensing

The determination of pH at the cellular level is of great importance to solve the concerned questions in biomedical research and it is still challenging to fabricate suitable pH probes for pH determination under NIR excitation. The luminescence-based pH sensors depend on the employment of nanomaterials that respond to pH with the change in their light emission properties. Recently, studies on UCNP-based pH-sensors have come within striking distance, and the first review in the field was reported by Mahata et al. [154]. A UCNP-based pH probe was initially presented by the group of Wolfbeis in 2009 [155]. They designed a sensor film by using NaYF_4_: Er, Yb nanorods and a pH indicator (bromothymol blue) with a biocompatible polyurethane hydrogel. The pH probe exhibited a large spectral shift and color change with different values of pH.

**Figure 10:**
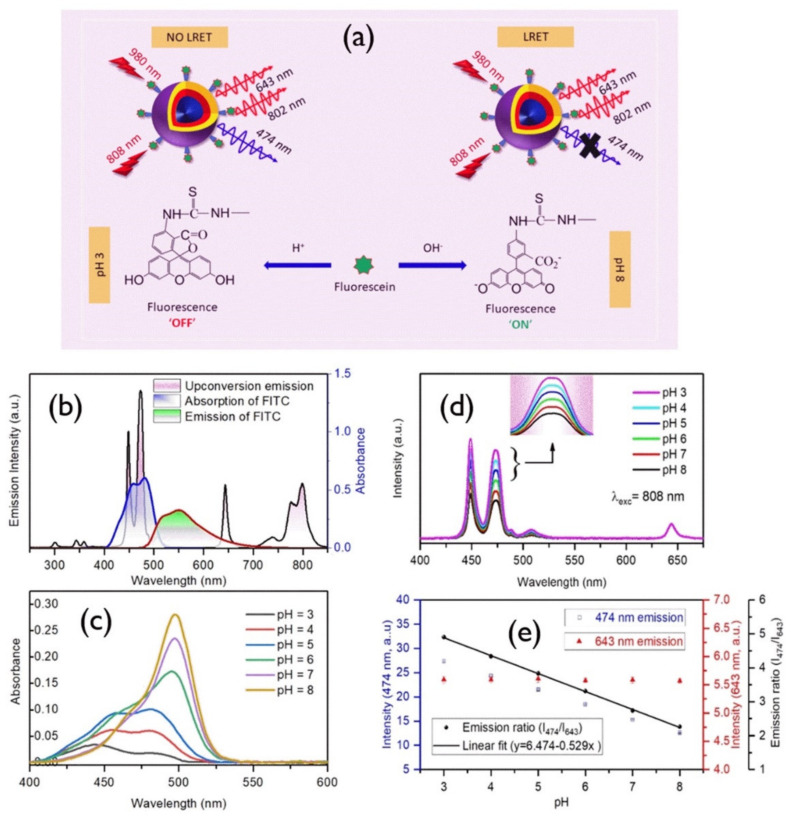
(**a**) Schematic description of the developed nanoprobe and its working principle. (**b**) Black line, upconversion emission upon 980 nm; blue line, absorption spectrum of FITC; red line, FITC emission upon 488 nm laser excitation. (**c**) Absorption spectra of FITC in various pH values. (**d**) Variation of 808 nm excited upconversion emission spectra of FITC-conjugated core–shell–shell nanoprobes with pH; inset shows the 474 nm band. (**e**) Variation of 474 nm, 643 nm and their ratio (474 to 643 nm) with pH values ranging from 3 to 8 [157].

A ratiometric pH sensor was developed by using NaYF_4_:Yb, Er UCNPs and porphyrin derivatives [156]. In this report, Esipova et al. established that by monitoring the change in the red/green ratio of upconversion emission, the pH can be detected. Mahata et al. [157] recently reported a rationally designed upconversion system (Figure 10) that combines NaYF_4_:Yb^3^^+^/Tm^3^^+^@NaYF_4_:Yb^3^^+^@NaYF_4_:Yb^3^^+^/Nd^3^^+^ with fluorescein-5-isothiocyanate (FITC) and its pH sensing upon 980 and 808 nm laser light excitations (Figure 10). The Tm^3^^+^ upconversion luminescence bands in the blue region were found to vary in pH because of the absorption of blue-upconverted light by the conjugated dye molecules, while the other luminescence bands at the red or near-infrared regions remained unchanged. Thus, a self-referenced ratiometric sensor was designed by using the 474 nm, 643 nm, and 802 nm upconversion emissions upon longwave light excitations. This approach will open pH sensing in the biomedical field [157].

Recently, the Hirsh and Hall group have jointly developed a pH framework using UCNPs and charged dyes based on their spectral overlap [158]. This method investigated the coupling of two anthraquinone dyes, Alizarin Red S and Calcium Red, at various pH values, where the green upconversion band was reduced by an inner filter effect and the red emission band remained unchanged. In this study, the overlap of the green emission with the upconversion spectrum and absorption spectra of Calcium Red at various pH values, validated the design of the pH sensor.

## 4. Conclusions and Perspectives

Recently, research on UCNPs’ biological applications with in vivo and in vitro studies have contributed many breakthroughs in various fields, including therapy, immune regulation, imaging, visual neurophysiology and optogenetics, suggesting their potential biomedicine applications in the immediate future. The progress on UCNP research has already established a strong base and more researchers are involved in the related field from physics, chemistry, and biology. However, most of the studies present the feasibility of UCNPs in biological applications through live-cell or small animal imaging, but they are still unsuitable for clinical practice. Some major challenges that should be focused on in UCNP research are as follows: (1) quantum efficiency of the UCNPs should be increased—although some strategies have been undertaken, further research is required—, (2) surface modification for biointerfacing of the nanoparticles, and (3) preclinical studies should be extended to large animals. Researchers also need to consider how to use UCNPs as detection reagents in molecular biology as well as nanodrug carriers in drug-delivery. Collaborative efforts are important from physicists, chemists and biologists to improve UCNP aspects in biomedical research. However, biosafety of the nanoparticles should be assessed competently prior to their clinical trials. Investigations on the long-term use of UCNP should be conducted, whereas most of the reports at present are based on short-term findings. These studies should exclusively explore the ROS generation, immune therapy for tissues and organs, levels of molecular metabolism, and protein expression. Cytotoxicity, excretion of the UCNPs, and their bio-distribution are very important parameters for UCNPs’ biological applications. The UCNPs injected into the body should be cleared thoroughly within a specific time. It is reported that most of the drugs are removed from the body by biliary or renal excretion. Foreign nanoparticles are cleared through phagocytosis. Some reports present that after 115 days of UCNP injection, they are completely removed from the body of mice, whereas for bigger UCNPs, it may take years to clear from the body. Until now, their excretion mechanism is not completely understood, and systematic data are required to obtain exact information of UCNPs’ biological effects. 

Apart from these, many aspects associated with UCNPs remain as a great concern, such as surface modification, efficiency enhancement, emission color tuning, and manipulation of energy transfer pathways for specific applications. Very recently, upconversion nanoparticles have been studied to optically stimulate the brain of the mammalian system by activating the light-gated ion channels. However, one of the major challenges is the design of the potent nanoparticles is so that they can be (a) bright, (b) photostable, (c) long-term function capable, and (d) non-toxic. Specifically, in order to design effective nanostructures, the major problems to understand the effect of energy migration dynamics on the temporal behavior of upconversion emission and ambiguous recognition of the energy migration dynamics should be resolved.

Nanoparticles do not need gene therapy; thus, their clinical translation can be safely and easily controlled. UCNPs can be recommended to nanopharmaceutical regulatory authorities. However, some certain issues persist ahead of clinical approaches. For example, pro-inflammatory activity and biocompatibility with neural tissues must be invesigated; UCNPs fate, including the clearance and diffusion from the injection location, needs to be assessed. The encapsulation, degradation and inactivation in the cellular environment need to be explored. For optogenetics, UCNPs’ exit from the brain and their accumulation in the peripheral organs may cause significant side effects which should be minimized. Apart from that, for energy transduction and neurostimulation, the interface between UCNPs and nervous tissue is a potential factor. A good balance is essential between the toxicity and brightness for successful clinical translation.

Due to the safety consideration, the applications of UCNPs (in vitro and in vivo) are limited to laboratory use. Probable toxic effects may emerge from the UCNPs’ chemical composition. The size of the UCNPs should be optimized on the basis of their interactions with cells, which demands a completely reproducible and standard synthesis method, along with suspension stability in biological media over time at 37 °C. The end users should comply with proven safety data. Therefore, to fill the laboratory to clinical translational gap, a risk–benefit assessment of theranostic applications is fundamentally required.

To conclude, biomedicine applications of UCNPs have excellent possibilities, but they have several challenges. We anticipate that the cooperative efforts of scientists will make a broad and bright future in its prospect and pave the way in the right direction for the prospects of the UCNPs’ biological applications.

## Figures and Tables

**Table 1:** Upconversion emission of various RE-doped materials.

Host Material	Excitation Wavelength (nm)	Dopant Ions	Major Emission Bands			Reference
			Blue	Green	Red	
CaMoO_4_	980 nm	Yb^3^^+^/Er^3^^+^	475	530, 552	656	[32]
NaYF_4_	980 nm	Yb^3^^+^/Tm^3^^+^	460, 477		650	[33]
NaGdF_4_	980 nm	Ho^3^^+^/Yb^3^^+^	487	541	647, 751	[35]
Y_2_O_3_	980 nm	Yb^3^^+^/Er^3^^+^	487	522, 554	664	[36]
LaF_3_	980 nm	Yb^3^^+^/Tm^3^^+^	475		800	[38]
LaF_3_	980 nm	Yb^3^^+^/Er^3^^+^		521, 545	660	[40]
Na_2_Y_2_B_2_O_7_	980 nm	Yb^3^^+^/Er^3^^+^	476, 488	525, 550	660	[39]
YVO_4_	980 nm	Yb^3^^+^/Er^3^^+^		525, 554	661	[37,45]
YVO_4_	980 nm	Yb^3^^+^/Ho^3^^+^		538, 548	655	[46,47]
BaTiO_3_	980 and 800 nm	Yb^3^^+^/Er^3^^+^	476	563	650	[41,48]
BaTiO_3_	980 nm	Yb^3^^+^/Ho^3^^+^		538, 548	655	[34]
Gd_2_Mo_3_O_9_	980 nm	Yb^3^^+^/Er^3^^+^		527, 547	660	[49]

**Table 2:** Some examples of UCNPs and their biomedicine applications along with excitation and emission wavelengths.

UCNP	Excitation/Emission Wavelength (nm)	Application	Reference
NaLuF_4_:Yb^3+^,Tm^3+^@SiO_2_-GdDTPA	980/800	T_1_ MR, CT and NIR-I imaging	[70]
LiYF_4_:Yb^3+^/Tm^3+^@SiO_2_@GPS@CH/PhL/PEGBA	980/792	Drug delivery and NIR-I imaging	[71]
NaYbF_4_:2%Tm^3+^	980/800	CT and NIR-I imaging	[72]
NaYF_4_:Yb^3+^,Tm^3+^@Fe_x_O_y_	980/800	T_2_ MR, CT and NIR-I imaging	[73]
CaF_2_:Tm^3+^,Yb^3+^	920/800	NIR-I imaging	[74]
NaYF_4_:Nd^3+^@NaLuF_4_@PDA_18_	808/1060	NIR-II, CT imaging and PTT	[75]
NaYF_4_:Yb,Nd@CaF_2_-PAA	808/980, 1350	Multiplexed NIR imaging	[76]
NaErF_4_:2%Ho@NaYF_4_	1530/980, 1180	Biosensing	[77]
Nd^3+^: LaF_3_	808/900, 1060	Sub-tissue thermal sensing	[78]
NaYbF_4_:Tm^3+^	915/800	NIR-I imaging	[79]
NaDyF_4_:10%Nd-GA-Fe	808/1050	NIR-II, T_2_ MR imaging and PTT	[80]
NaYF_4_:Yb,Ho/Pr/Tm/Er@NaYF_4_	980/1185/1310/1475/1525	Disease-targeted NIR-II imaging	[81]
NaGdF_4_:Yb,Er@NaGdF_4_:Nd,Yb-RB	808/970	T_2_ MR, NIR imaging and PDT	[82]
